# Identification of powdery mildew resistance quantitative trait loci in melon and development of resistant near-isogenic lines through marker-assisted backcrossing

**DOI:** 10.1186/s40529-024-00435-x

**Published:** 2024-11-04

**Authors:** Chun-San Wang, Ssu-Yu Lin, Jin-Hsing Huang, Hsin-Yi Chang, Di-Kuan Lew, Yu-Hua Wang, Kae-Kang Hwu, Yung-Fen Huang

**Affiliations:** 1 Department of Agronomy, National Taiwan University, No. 1, Sec. 4, Roosevelt Rd., Da’an Dist., Taipei City, 106319 Taiwan; 2 Crop Genetic Resources and Biotechnology Division, Taiwan Agricultural Research Institute, Ministry of Agriculture, No. 189, Zhongzheng Rd., Wufeng Dist., Taichung City, 413008 Taiwan; 3 Plant Pathology Division, Taiwan Agricultural Research Institute, Ministry of Agriculture, No. 189, Zhongzheng Rd., Wufeng Dist., Taichung City, 413008 Taiwan; 4 Crop Science Division, Taiwan Agricultural Research Institute, Ministry of Agriculture, No. 189, Zhongzheng Rd., Wufeng Dist., Taichung City, 413008 Taiwan

**Keywords:** *Cucumis melo*, Melon, Powdery mildew, *Podosphaera xanthii*, Quantitative trait loci (QTL), Marker assisted backcrossing (MABC), TaqMan, Double digest restriction-site associated DNA (ddRAD) sequencing

## Abstract

**Background:**

Melon (*Cucumis melo* L.), an important cucurbit crop, faces production limitations due to powdery mildew (PM). Developing resistant varieties offers a sustainable, genetics-based alternative to chemical treatments. Therefore, identifying PM resistance quantitative trait loci (QTL) and creating trait-associated markers are essential for efficient melon PM resistance improvement through marker-assisted backcrossing (MABC).

**Results:**

Three F_2_ populations, A6, B2, and C4, were generated for QTL mapping of PM resistance. Major QTL were identified on chromosome 2 in A6, chromosome 5 in B2, and chromosomes 5 and 12 in C4. A series of TaqMan® assays targeting regions on chromosomes 2, 5, and 12 were developed and validated for foreground and recombinant selection, complemented by the double digest restriction-site associated DNA genotyping system to evaluate the recurrent parent genome recovery. Three MABC programs using resistant donor parents from A6 and C4 crossed with elite susceptible recurrent parents with green and orange fruit flesh were implemented. After two to three cycles of MABC, individual QTL was successfully introgressed into elite genetic backgrounds, giving six PM resistance lines in each green- and orange-fleshed background. PM inoculation on the twelve near-isogenic lines confirmed their resistance to PM.

**Conclusions:**

We have identified major PM resistance QTL for melon on chromosomes 2, 5, and 12 and have introgressed individual QTL to elite genetic backgrounds using MABC in three and a half years. This study demonstrates the power of combining high-throughput genotyping with breeding efforts and showcases the efficiency of molecular breeding.

**Supplementary Information:**

The online version contains supplementary material available at 10.1186/s40529-024-00435-x.

## Introduction

Melon (*Cucumis melo* L., 2n = 2x = 24) is an economically valuable crop in the Cucurbitaceae family, cultivated widely across the world from temperate to tropical regions due to its sweet taste, flavor, and fleshy fruit. The global production of melons was around 28.56 million tonnes, making it the third most-produced cucurbit crop after watermelon and cucumber (FAOSTAT 2022). Melon is a cross-pollinated species and is an important model plant for studying sex determination. Initially, melon flowers are bisexual but can undergo abortion of either the carpel or stamen during sex determination, leading to the development of andromonoecious (producing male and bisexual flowers) or gynomonoecious (producing female and bisexual flowers) plants (Aamir et al. 2021). Typically, commercially grown melons are monoecious or andromonoecious, with male flowers on the main stem and female or hermaphrodite flowers on the proximal nodes of lateral branches. Consequently, melon breeding programs must initially assess self-pollinated breeding lines before proceeding to evaluate the hybrids.

Powdery mildew (PM) significantly impacts melon production, causing economic losses across all growing areas. This fungal disease can affect seedlings, stems, leaves, and fruit, which typically display a powdery appearance due to the abundance of conidia. It ultimately results in fruit quality degradation and yield losses due to stunting and premature plant death, limiting melon cultivation in greenhouses and fields (Egel et al. 2022). PM is caused by *Podosphaera xanthii* (Castagne) U. Braun & Shishkoff and *Golovinomyces cichoracearum* (DC.) V.P. Heluta. *P. xanthii* occurs more frequently in regions with high humidity and temperatures, such as subtropical and tropical areas. More than 20 races of *P. xanthii* have been identified based on the reactions to the differential set (McCreight 2006). Races 1, 2, and 5 are dominant races in Southern Europe; races 1 and 5 are prevalent in Japan; races 1, 2, and 3 in America; and races 1 and 2F are common in China (Zhang et al. 2013; Haonan et al. 2020). Races 1 and 5 were detected in Taiwan, with the former being predominant from 2001 to 2005, and the latter identified in 2005. From 2008 to 2010, both races were commonly identified in Tainan, the primary region for melon production in Taiwan (Huang and Wang 2007; Wang 2016). The distribution of predominant physiological races is influenced by spatiotemporal situations and the specific melon cultivars grown in these regions (López-Martín et al. 2022). PM management in melon involves cultural practices, biological controls, fungicides, and host resistance (Egel et al. 2022). Although fungicides are commonly used for melon PM control, they are not a sustainable solution since they can cause plant resistance breakdown and harm the environment. Therefore, resistant varieties remain a more sustainable and efficient option for PM control (Branham et al. 2021; Egel et al. 2022).

Identifying PM resistance quantitative trait loci (QTL) and the germplasm that carries them are essential for resistance breeding. Once the resistant materials and loci are identified, resistance loci can be effectively introduced into elite backgrounds using marker-assisted selection (Collard and Mackill 2008). Several studies have identified the genetic resources and QTL for PM resistance in melon. PM resistance QTL in melon have been identified on chromosomes 2, 4, 5, 9, 10, and 12 (Perchepied et al. 2005; Teixeira et al. 2008; Fukino et al. 2008; Yuste-Lisbona et al. 2010, 2011a, b; Wang et al. 2011, 2016; Beraldo-Hoischen et al. 2012; Zhang et al. 2013, 2023; Fazza et al. 2013; Ning et al. 2014; Kim et al. 2016; Li et al. 2017; Haonan et al. 2020; Cao et al. 2021; Branham et al. 2021; Cui et al. 2022; López-Martín et al. 2022). Regarding PM resistance materials, PI 414723, K7-1, and TARI-08874 were reported to carry a single QTL on chromosome 2 (Zhang et al. 2013; Fazza et al. 2013; Wang et al. 2016). The melon variety Ano2 carried a QTL on chromosome 5 (Wang et al. 2011), and the accession AF125^*Pm−1*^ carried resistance QTL on chromosome 9 (Teixeira et al. 2008), while the accessions wm-6, PI 124112, and MR-1 carried a single resistance QTL on chromosome 12 (Li et al. 2017; Cao et al. 2021; Zhang et al. 2023). Some accessions carried more than one resistance QTL. For instance, Edisto47 carried resistance QTL on chromosomes 2 and 5 (Ning et al. 2014); AR 5, PMR 5, and PMR 6 carried QTL on chromosomes 2 and 12 (Fukino et al. 2008; Kim et al. 2016; Haonan et al. 2020); and PI 124112 and TGR-1551 carried QTL on chromosomes 5 and 12 (Perchepied et al. 2005; Yuste‐Lisbona et al. 2010; Yuste-Lisbona et al. 2011a, b; Beraldo-Hoischen et al. 2012; López-Martín et al. 2022). Based on the abovementioned studies, the polymorphism of the PM markers are not able to correspond to the resistance consistently while applying marker-assisted selection, suggesting the investigation of high-density markers are required.

Next-generation sequencing (NGS) technologies and the release of the melon reference genome (Garcia-Mas et al. 2012) have revolutionized melon genetic analysis, offering faster methods to identify resistance QTL (Li et al. 2017; Branham et al. 2018). Genotyping-by-sequencing (GBS) techniques are a group of NGS-based high-throughput genotyping methods that use restriction enzymes to reduce genome complexity and dual-index barcoded systems to produce multiplex NGS libraries (Scheben et al. 2017). These methods include restriction site-associated DNA (RAD) sequencing (Baird et al. 2008), GBS (Elshire et al. 2011), 2-enzyme GBS (Poland et al. 2012), and double digest restriction site-associated DNA (ddRAD) sequencing (Peterson et al. 2012). NGS-based methods enable low-cost per-data-point marker discovery and high-throughput genotyping of single nucleotide polymorphisms (SNPs). Such availability enhances the construction of high-density linkage maps for QTL mapping and supports marker-assisted selection.

Marker-assisted backcrossing (MABC) utilizes the association between target phenotype and marker genotype for indirect selection (Frisch 2004). MABC has enhanced desirable characteristics in various crops, including submergence tolerance and salinity tolerance in rice (Neeraja et al. 2007; Marè et al. 2023), nutrient enrichment in maize (Singh et al. 2021; Chandrasekharan et al. 2022), and heat tolerance and fusarium head blight resistance in wheat (Zhang et al. 2021; Bellundagi et al. 2022). MABC aims to introgress favorable alleles from the donor parent into the recurrent parent’s genetic background. It involves three stages: foreground, recombinant, and background selection. Foreground and recombinant selections target the QTL-carrier chromosome with the objective of introducing an allele of interest with minimal linkage drag, while background selection aims to recover the recurrent parent genome (RPG) outside the target locus as much as possible (Collard and Mackill 2008). The effectiveness of MABC relies on adequate population size, the presence of polymorphic markers close to the target locus (Frisch et al. 1999a), and a broad distribution of markers within the genetic background (Frisch et al. 1999b). In melon, there have been cases of using markers to target disease resistance traits in backcross programs (Sousaraei et al. 2018; Palomares-Rius et al. 2018), but the implementation of MABC using markers for foreground, recombinant, and background selection has not yet been reported in the literature.

In this study, we aim to identify PM resistance QTL in the melon germplasms using high-density linkage maps, providing accurate markers for PM resistance. The objective of this study was (1) to develop high-density markers using an NGS-based high-throughput marker system, (2) to identify PM resistance QTL in melon, and (3) to introgress resistant alleles into elite melon cultivars using MABC. It took three and a half years from marker development to QTL introgression, which demonstrated the effectiveness of combining an NGS-based marker system and traditional breeding for the rapid improvement of disease resistance in melon.

## Materials and methods

### Plant materials

#### Three F_2_ populations for QTL mapping

In this study, we used three F_2_ populations, A6, B2, and C4, to map PM QTL (Table 1). The A6 population, composed of 165 F_2_ individuals, was derived from a cross between TARI-18-437 and TARI-18-494. The B2 population, composed of 179 F_2_ individuals, was derived from a cross between TARI-18-410 and TARI-18-491, while the C4 population, with a population size of 179 F_2_ individuals, was derived from a cross between TARI-18-491 and TARI-18-449. B2 and C4 shared TARI-18-491 as a common parent, but it served as the male parent for B2 and the female parent for C4.

**Table 1:** The parental lines of the F_2_ population and the MABC breeding programs

Population	Female parent	Male parent
*F*_*2*_ * populations for PM QTL mapping*		
A6	TARI-18-437	TARI-18-494
B2	TARI-18-410	TARI-18-491
C4	TARI-18-491	TARI-18-449
*Marker-assisted backcrossing*		
*qPM2*	TARI-18-432 TARI-18-431	TARI-18-437
*qPM5* and *qPM12*	TARI-18-432 TARI-18-431	TARI-18-449

TARI-18-432 and TARI-18-431 are the recurrent parents with green (G) and orange (O) flesh colors. The TARI-18-437 and TARI-18-449 are the PM resistance donor parents

The parental inbred lines, TARI-18-437 and TARI-18-410, were characterized by netted-rind, green-fleshed fruit, and resistance to *P. xanthii* race 1. TARI-18-437 was derived from a hybrid variety, and TARI-18-410 was generated from another accession after 16 and 14 generations of selfing, respectively. TARI-18-449 was a PM resistance inbred line developed through five generations of selfing of a hybrid variety with PI 124111 and PI 124112 in its pedigree. The PM resistance of the parental lines for the three F_2_ populations is shown in Table 2.

**Table 2:** The average DI of PM for the parental lines

Line	PM DI	
	2017	2018
TARI-18-437	0.0	0.0
TARI-18-494	6.8	7.3
TARI-18-410	1.3	0.0
TARI-18-491	7.5	6.8
TARI-18-449	0.0	0.0
TARI-18-432	6.5	7.5
TARI-18-431	2.5	5.8

#### The recurrent parents for MABC

Marketing preferences for new melon varieties primarily depend on fruit quality traits, particularly flesh color. Thus, we selected TARI-18-431, with orange flesh and a globular shape, and TARI-18-432, with green flesh and a rounded shape, as the elite recurrent parents (Table 1). Both parents possess desirable horticultural traits for fruit rind netting and taste quality but without PM resistance (Table 2). Therefore, the MABC breeding programs in this study aimed to improve the PM resistance of these two elite inbred lines by using them as recurrent parents.

### PM evaluation

The PM reaction was assessed using leaf disc inoculation, as described by Wang et al. (2016). The experimental unit consisted of two leaf discs of 15-mm diameter per genotype, sampled from the second leaf when the melon plant was at the third-leaf stage. The leaf discs were placed on M-solution (10,000 ppm mannitol, 30 ppm benzimidazole, and 50 ppm tetracycline) in 60-mm Petri dishes. In each Petri dish, IRANH, a susceptible variety, and PMR 45, a resistant variety, were used as experimental controls. For each genotype, the disease reaction was evaluated using two replications. A conidial suspension was uniformly sprayed over the leaf discs to a density of 50–100 spores per cm^2^. Leaf discs were incubated at 24 °C/18 °C (day/night) with a 12-hour photoperiod for 12 days. The disease index (DI) was scored for each disc on a scale of 0 to 9, where 0 = no lesions; 1 = lesions covering 10% of the leaf area; 3 = lesions covering 50% of the leaf area; 5 = lesions covering 80% of the leaf area; 7 = lesions covering 100% of the leaf area, with thin spores on the leaf; and 9 = lesions covering 100% of the leaf area, with a thick brown disc of sporangia on the leaf discs. Plants with a mean DI < 3.0 were considered resistant, while those with a mean DI ≥ 3.0 were susceptible (Huang et al. 2002). For each batch of experiments, a non-inoculated set was set aside as a negative control. Disease rating was conducted in the fungal disease laboratory of the Plant Pathology division, Taiwan Agricultural Research Institute (TARI), Taichung, Taiwan, in spring 2017 and autumn 2018. Based on the leaf disc PM reaction evaluation on the melon differential set (McCreight 2006), the physiological race inoculated in this study is *P. xanthii* race 1.

### Genotyping using ddRAD sequencing and its bioinformatic analysis workflow

#### ddRAD library preparation

Prior to library construction, we used *in silico* analysis on the melon reference genome DHL92 v3.5.1 (Argyris et al. 2015) and organelle genomes (Rodríguez-Moreno et al. 2011) to estimate adapter amounts and predict digested fragment sizes. Based on this analysis, the appropriate restriction enzyme sets for this study were *Pst*I, *Taq*^α^I, and *Sph*I. *Pst*I and *Taq*^α^I, serving as rare and common cutters, have average recognition site distances of 11,914 bp and 482 bp, respectively, which determined the adapter input amounts. *Sph*I was used to exclude target fragments containing the recognition site associated with rDNA sequences based on results from prior experiments.

Genomic DNA was extracted from freeze-dried young leaves using a modified 1.25% SDS method (Jobes et al. 1995). Extracted DNA was purified using the QIAquick 96 PCR Purification Kit (Qiagen, Hilden, Germany). DNA concentration was quantified using Quant-iT™ PicoGreen™ dsDNA Assay Kits (Life Technologies, Oregon, USA) and adjusted to 10–15 ng/µL for library preparation. The ddRAD library was prepared according to Peterson et al. (2012) with minor modifications as follows. The restriction enzymes, T4 DNA ligase, CutSmart Buffer, and rATP used for library construction were from New England Biolabs, Ipswich, MA, USA. Initially, 300 ng of genomic DNA from each sample was double-digested using *Pst*I-HF and *Taq*^α^I at 37 °C and 65 °C, respectively, for 30 min. Ligation was then proceeded overnight at 16 °C, incorporating a 5-fold excess of P1 (*Pst*I-HF) and Y (*Taq*^α^I) adapters, T4 DNA ligase, and rATP in 1X CutSmart Buffer. After inactivating the ligase, the library was treated with *Sph*I at 37 °C for 30 min to exclude sequences and then purified using 0.8X AMPure XP beads (Beckman Coulter, Brea, CA, USA) to eliminate short fragments and adapter dimers. PCR amplification was conducted using dual-indexed primers, including Nextera XT DNA Indexes v2 and Phusion polymerase (Thermo Fisher Scientific Baltics UAB, Vilnius, Lithuania) in a 20-cycle two-step PCR protocol, which included only the denature and extension steps. The final PCR products were purified using 0.8X AMPure XP beads, quantified with Quant-iT™ PicoGreen™ dsDNA Assay Kits, and pooled in equal amounts. The ddRAD library target fragments were size-selected, ranging from 300 to 600 bp, using Blue Pippin (Sage Science, Beverly, MA, USA) in a 2% electrophoresis gel cassette. The size-selected ddRAD libraries were then sent to the Core Facility of the Cancer Progression Research Center at National Yang Ming Chiao Tung University for PE250 sequencing with extra dark cycles and a spike-in of 10% PhiX, utilizing the HiSeq2500 Rapid mode (Illumina, San Diego, CA, USA).

#### Bioinformatic analysis workflow for SNP calling

Paired-end FASTQ files were processed with AdapterRemoval v2.1.7 (Schubert et al. 2016) and aligned to the DHL92 v3.5.1 reference genome using Bowtie2 v2.2.9 (Langmead and Salzberg 2012). Alignments were sorted, compressed, and indexed into BAM files with SAMtools v1.3 (Li et al. 2009), and optical duplicate reads were removed using PICARD tools (http://broadinstitute.github.io/picard). Following GATK v3.8 (McKenna et al. 2010) Best Practices recommendations, base quality score recalibration refined sequence quality scores through a recalibration model. This model, typically relying on a known variants database such as dbSNP to distinguish true genetic variants from sequencing errors, utilized VCF files from HaplotypeCaller of parental BAM files as a dbSNP substitute, improving variant calling accuracy. F_2_ population BAM files were recalibrated, filtered with FilterSamReads, and then converted to gVCFs using HaplotypeCaller. Finally, aggregating all gVCFs through GenotypeGVCFs produced the population’s VCF file (DePristo et al. 2011; Van der Auwera et al. 2013).

The raw VCF files were subjected to filtering with specified criteria: (1) variants with more than two alleles were removed; (2) variants with a QUAL score less than 200, a Quality by Depth (QD) less than 10, and a minor allele frequency under 0.01 were excluded; and (3) genotypes with a Genotype Quality (GQ) from the VCF file’s FORMAT fields below 20 were marked as missing, increasing the missing rate threshold to 0.25.

### Linkage map construction and QTL mapping

For linkage map construction and QTL mapping, we used the R/qtl package (Broman et al. 2003) implemented in R software (version 4.3.1, R Core Team 2023). Within each F_2_ population, the segregation distortion of each SNP was tested using the *χ*^*2*^ goodness-of-fit with a threshold adjusted by Bonferroni correction (*α* = 0.05/number of SNPs). SNPs were ordered based on physical positions, marker phasing was verified, and recombination fractions were estimated. Upon visual inspection of the recombination fraction patterns along the chromosomes, SNPs that were physically close to each other but showed no linkage were removed. Additionally, the SNP order was manually refined based on the recombination fraction patterns or physical positions, as necessary.

To prevent linkage map expansion frequently encountered in high-density maps, the Genotype Corrector (Miao et al. 2018) was applied to identify genotype errors and impute missing genotypes. Genotype Corrector used a sliding-window algorithm. The window size was set to 13 after empirical tests with the data from this study. The linkage map was then re-estimated using the Kosambi mapping function based on the corrected and imputed genotype data.

Both single QTL analysis and multiple interval mapping (Manichaikul et al. 2009) based on Haley and Knott regression (Haley and Knott 1992) were used for QTL mapping. For single QTL analysis, the empirical threshold was determined for each population based on 1,000 permutations (Churchill and Doerge 1994) at *α* = 0.05. For multiple interval mapping, 1,000 permutations for pairwise QTL mapping were first performed for each population. Thresholds for individual QTL and pairwise epistasis were derived from the permutation results at *α* = 0.01 with heavy interaction penalties only.

#### Conversion between physical and genetic distances

To integrate the relationship between genetic distance and physical distance from the three F_2_ populations, the locally estimated scatterplot smoothing (LOESS) regression was employed. The regression model for each chromosome used 10% local neighborhood data points and incorporated a quadratic term (degree of 2) in the fitting predictors (Siberchicot et al. 2017). The LOESS regression model on the carrier chromosome was used to select candidate SNPs for foreground and recombinant selection.

### TaqMan^®^ assay development for MABC

Prior to designing TaqMan^®^ assays, MABC parental lines were genotyped using ddRAD sequencing. Polymorphic SNPs on carrier chromosomes with high quality (QUAL > 1000 and QD > 10) were selected. Each selected SNP was at least 50 bp away from any other variants and positioned at least 30 bp from the edge of the target fragments. Sequences of target fragments carrying the selected SNPs were then designed using the Custom TaqMan^®^ Assay Design Tool (Thermo Fisher Scientific 2024). The TaqMan^®^ assays for foreground selection were chosen based on the confidence intervals extending to the closest flanking markers. For recombinant selection, designed SNP markers were selected considering an average seed yield of approximately 200–500 per cross and the necessity of having at least one individual per cross for each MABC generation to manage risks effectively. The assays were chosen to cover at least 20 cM on both sides of the target region. TaqMan^®^ assays were validated using parental DNA samples. Recombinant and foreground selection were made using the TaqMan^®^ SNP Genotyping Assay Mix (Life Technologies, Marsiling, Singapore) and TaqMan™ Universal PCR Master Mix II, no UNG (Thermo Fisher Scientific Baltics UAB, Vilnius, Lithuania) according to the manufacturer’s instructions.

### MABC for PM resistance introgression

**Fig. 1:**
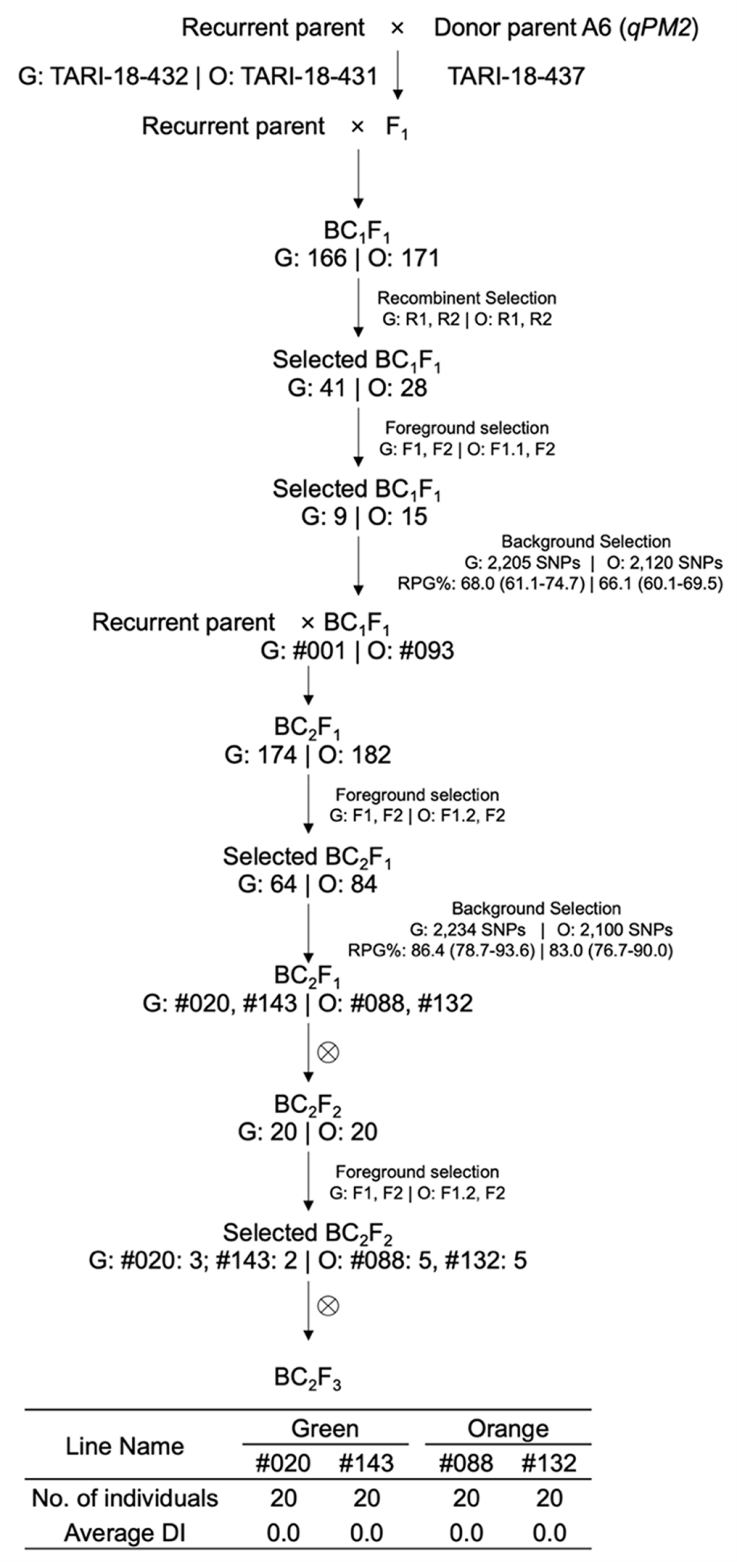
MABC scheme for *qPM2*. Crosses were made between the donor TARI-18-437 and recurrent parents TARI-18-432 (green-fleshed, G) and TARI-18-431 (orange-fleshed, O). G: Number | O: Number indicates the number of individuals for the green- and orange-fleshed background at each step. R1, R2, F1, F1.1, F1.2, and F2 are markers used for recombinant and foreground selection (Table 5). #Numbers indicate the selected line names derived from the selected individuals. Background selection text boxes contain the number of SNPs for RPG evaluation and average RPG recovery (%) with their ranges in brackets under green- and orange-fleshed backgrounds

**Fig. 2:**
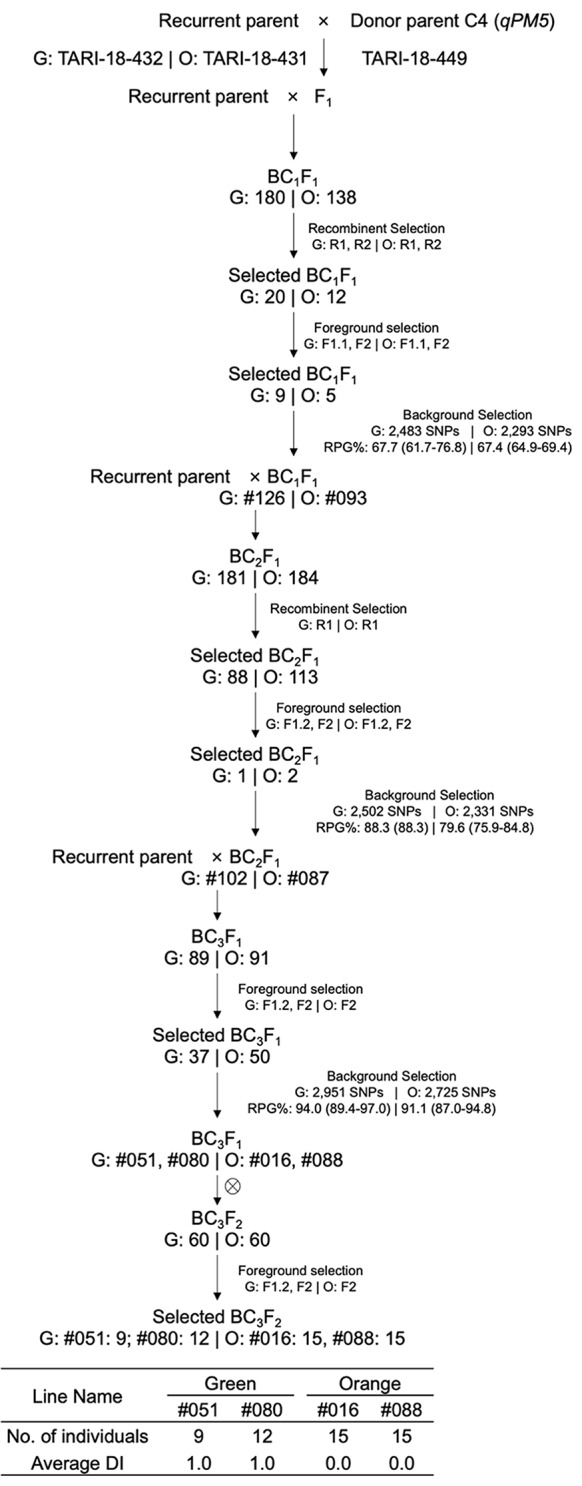
MABC scheme for *qPM5*. Crosses were made between the donor TARI-18-449 and recurrent parents TARI-18-432 (green-fleshed, G) and TARI-18-431 (orange-fleshed, O). G: Number | O: Number indicates the number of individuals for the green- and orange-fleshed background at each step. R1, R2, F1, F1.1, F1.2, and F2 are markers used for recombinant and foreground selection (Table 5). #Numbers indicate the selected line names derived from the selected individuals. Background selection text boxes contain the number of SNPs for RPG evaluation and average RPG recovery (%) with their ranges in brackets under green- and orange-fleshed backgrounds

**Fig. 3:**
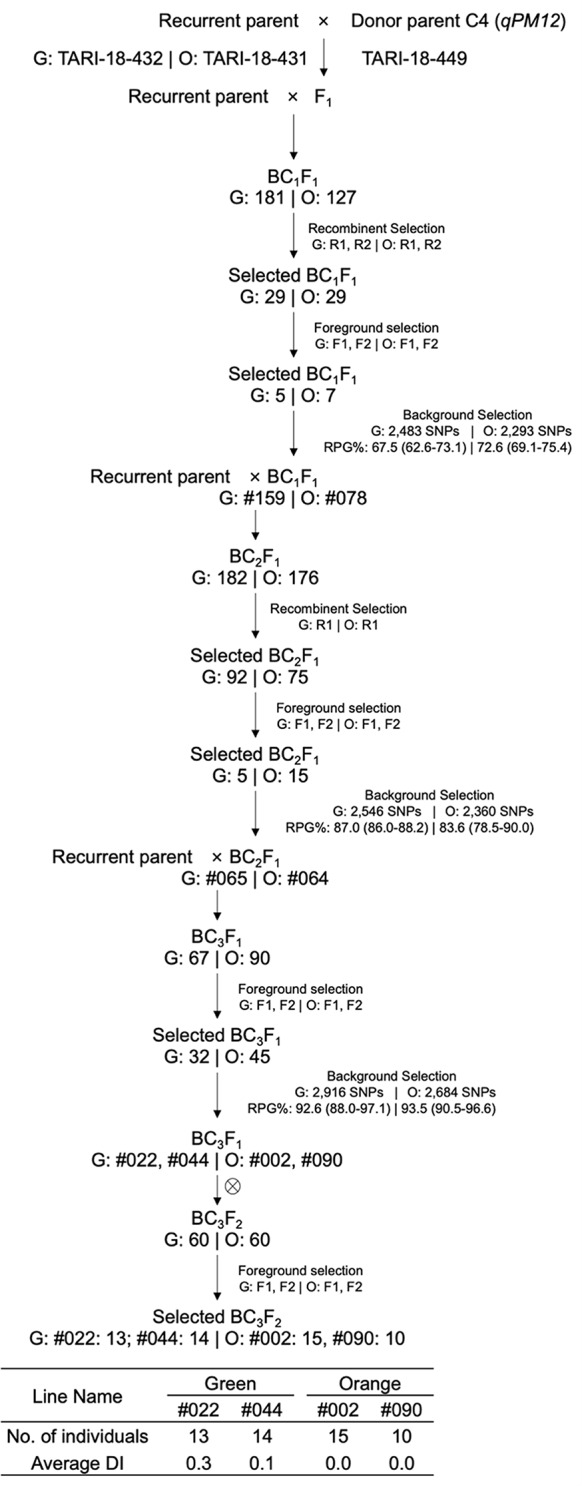
MABC scheme for *qPM12*. Crosses were made between the donor TARI-18-449 and recurrent parents TARI-18-432 (green-fleshed, G) and TARI-18-431 (orange-fleshed, O). G: Number | O: Number indicates the number of individuals for the green- and orange-fleshed background at each step. R1, R2, F1, F1.1, F1.2, and F2 are markers used for recombinant and foreground selection (Table 5). #Numbers indicate the selected line names derived from the selected individuals. Background selection text boxes contain the number of SNPs for RPG evaluation and average RPG recovery (%) with their ranges in brackets under green- and orange-fleshed backgrounds

The MABC aimed to enhance PM resistance in two elite recurrent parents. The process involved crossing donor parents with recurrent parents and selecting offspring according to target regions designated as *qPM2*, *qPM5*, and *qPM12* (Figs. 1, 2 and 3).

At the BC_1_F_1_ generation, we aimed to first reduce the linkage drag around target loci via recombinant selection. Only individuals carrying homozygous for the recurrent parent at one recombinant marker and heterozygous genotypes at the other marker were passed to foreground selection. Those carrying resistance donor alleles showing heterozygous genotypes in two bracketed foreground markers were passed to background selection. Background selection was performed using ddRAD sequencing to well cover the whole genome, and the individuals showing high RPG recovery were then crossed with the recurrent parent to produce BC_2_F_1_ generation. The RPG recovery of the selected individuals was estimated using ddRAD genotype data. Heterozygous genotypes were assigned a value of 0.5, and homozygous genotypes of the recurrent parent were assigned a value of 1. These values were then weighted by the genetic distance between flanking markers and divided by the total genetic distance covered by the ddRAD markers on the non-carrier chromosomes.

At the BC_2_F_1_ generation, recombinant selection was first applied to screen for homozygous individuals at the second recombinant marker. Individuals showing homozygous genotypes at both recombinant markers were kept for foreground selection. Heterozygous individuals at foreground markers were selected for background selection using ddRAD sequencing. Individuals with the highest RPG recovery were backcrossed to the recurrent parents to generate BC_3_F_1_ individuals.

At the BC_3_F_1_ generation, only foreground selection was conducted. Subsequently, individuals with the highest RPG, as determined through ddRAD sequencing, were selected and self-pollinated to produce BC_3_F_2_ progeny. In the final BC_3_F_2_ generation, foreground selection was conducted to identify candidate individuals carrying homozygous PM resistance alleles. These individuals were then self-pollinated to establish the near-isogenic lines as the final outcome of the MABC in this study. The MABC schemes, as generally outlined above, were subject to minor adjustments depending on specific circumstances.

## Results

### The DI of PM in three F_2_ populations

**Fig. 4:**
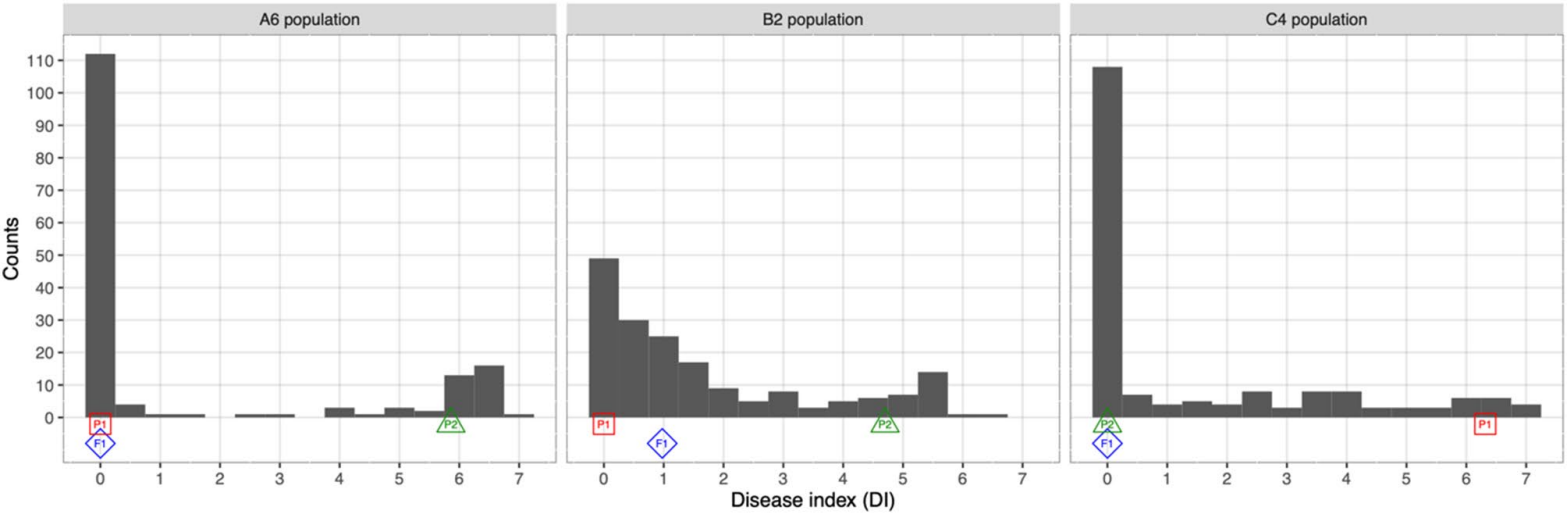
The PM DI distribution of the three F_2_ populations. The average DI of the two parents and F_1_ are indicated below the histogram using red square, green triangle, and blue diamond, respectively

The DI of PM in melon for the A6, B2, and C4 populations, their parental inbred lines, and F_1_ individuals are shown in Fig. 4. Disease reactions of most of the F_2_ individuals were between those of the two parents for the three populations. The F_1_ individuals showed a resistant phenotype, indicating that PM resistance was a dominant trait in the three populations. The phenotypic distribution of each F_2_ population is largely skewed toward resistance, suggesting that PM resistance is controlled by a few major QTL in the populations of interest.

### PM resistance QTL

**Fig. 5:**
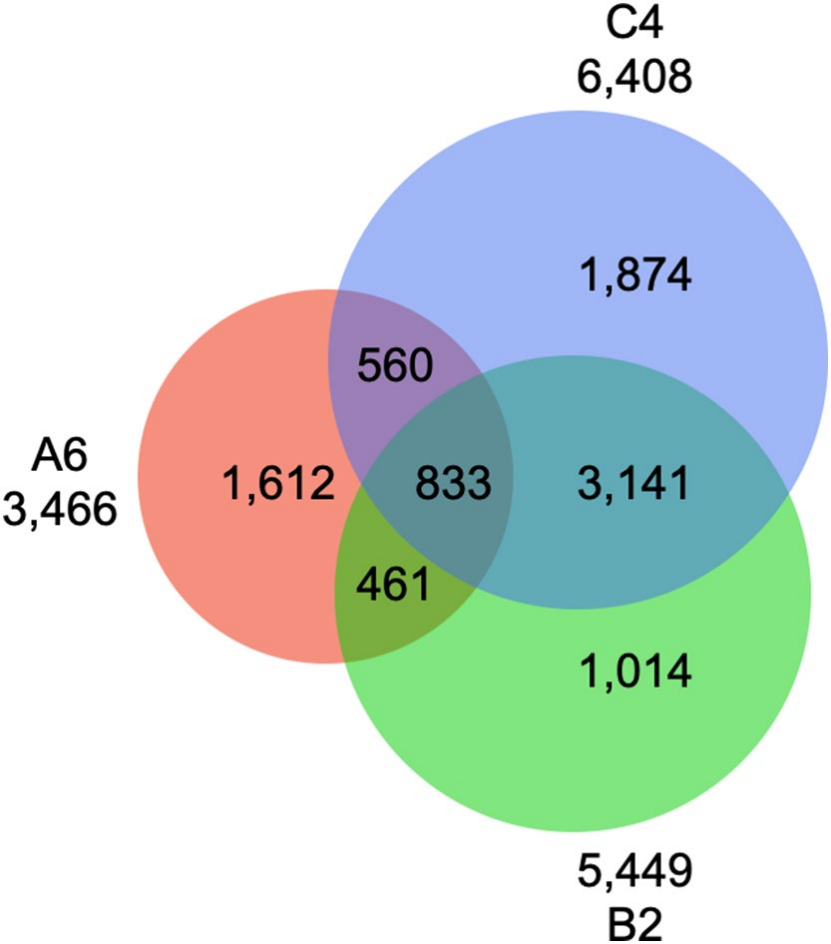
Number of SNPs across the three F_2_ populations

**Fig. 6:**
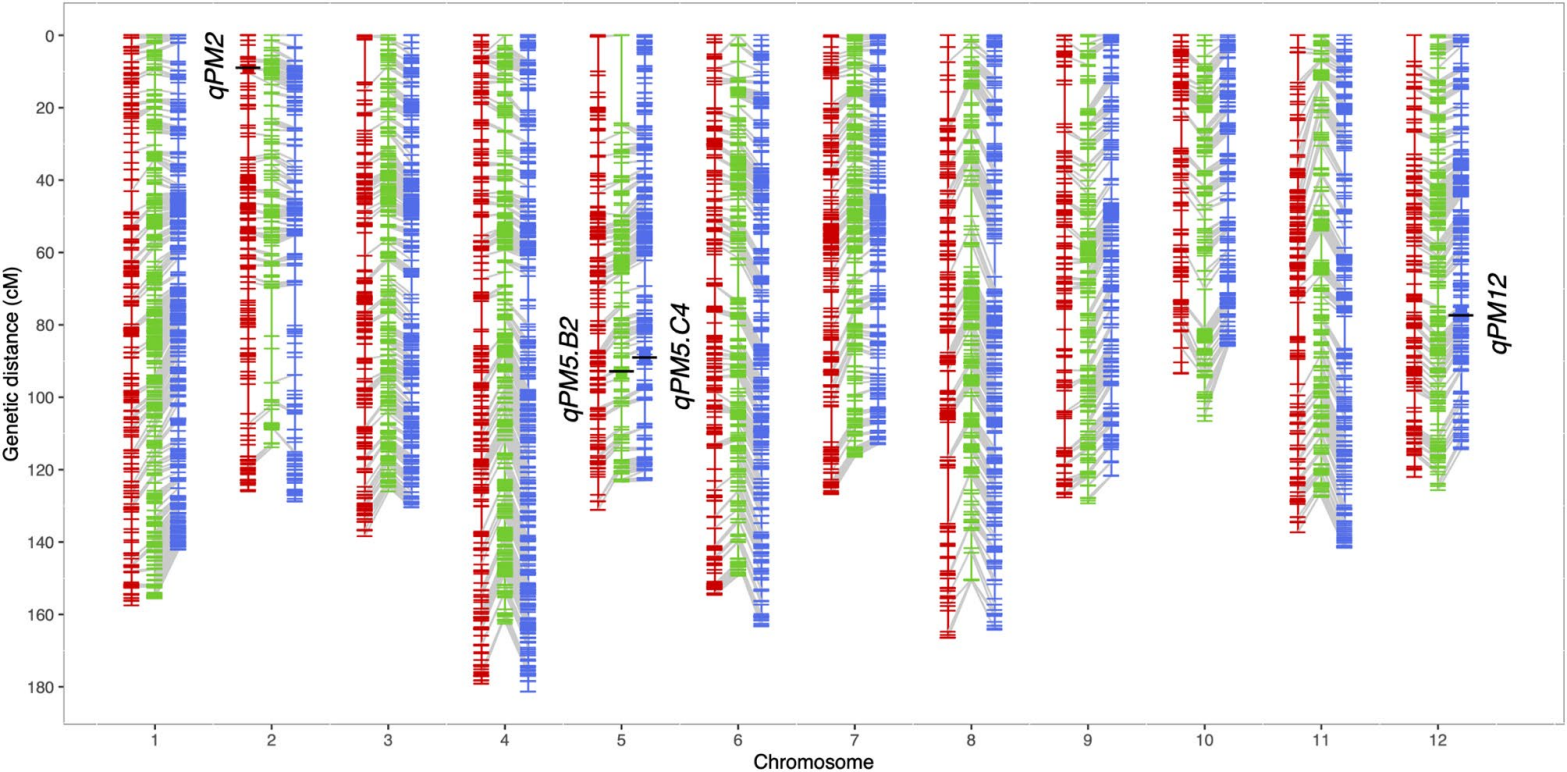
The linkage maps of the three F_2_ populations. The three F_2_ populations, A6, B2, and C4, are shown in red, green, and blue, respectively. The common markers between populations are linked using gray lines, and the black bars on the linkage groups are the QTL identified in this study. *qPM5.B2* and *qPM5.C4* represent the *qPM5* detected in B2 and C4 population, respectively

For A6, B2, and C4, 127.14, 135.97, and 138.65 million paired-end reads were generated, respectively. On average, 0.76 million paired-end reads per sample were available for the three populations. The number of variants was 454,561 for A6, 444,854 for B2, and 465,971 for C4. After quality filtering, segregation distortion tests, and correction for genotyping errors, the final SNP data sets consisted of 3,466 SNPs for A6, 5,449 SNPs for B2, and 6,408 SNPs for C4. Across the full SNP data, 833 SNPs were shared by the three F_2_ populations, while B2 and C4 shared up to 73% or 62% common SNPs, given that the two populations had a common parent (Fig. 5). The total map lengths were 1,659.8 cM for A6, 1,586.0 cM for B2, and 1,609.2 cM for C4. SNPs were evenly distributed along the genome, except for some gaps on chromosomes 2 (B2, C4), 3 (A6), 5 (B2), 8 (A6), 9 (A6), and 10 (B2) (Fig. 6; Table 3).

**Table 3:** Summary of linkage maps used in this study

	No. of markers				Length (cM)				Max interval (cM)		
Chr.	A6	B2	C4		A6	B2	C4		A6	B2	C4
1	294	761	873		157.4	155.4	142.1		5.8	4.2	5.1
2	250	199	296		126.0	113.8	128.8		8.2	13.3	11.2
3	345	518	601		138.3	126.0	130.4		11.8	4.2	4.8
4	401	644	721		179.1	162.5	181.4		7.9	5.1	6.5
5	281	334	430		131.0	123.3	123.0		9.7	24.3	7.2
6	309	488	588		154.5	149.2	163.3		6.8	9.0	4.2
7	324	428	511		126.7	116.3	112.9		9.9	3.4	4.5
8	283	414	543		166.4	150.5	164.2		15.5	8.4	9.3
9	245	399	425		127.7	129.3	121.6		10.9	7.1	5.4
10	169	277	327		93.4	106.4	85.8		7.8	10.9	5.4
11	267	485	595		137.3	127.4	141.5		9.5	8.1	8.9
12	298	502	498		122.0	125.7	114.3		7.2	5.1	5.7
Overall	3,466	5,449	6,408		1,659.8	1,586.0	1,609.2		15.5	24.3	11.2

The QTL identified in this study are summarized in Table 4 and Fig. 6. One major QTL, *qPM2*, was identified in A6. It was located on chromosome 2 and explained up to 93% of the total phenotypic variance. In B2, one major QTL, *qPM5*, was identified on chromosome 5, explaining 80% of the phenotypic variance. Two QTL, *qPM5* and *qPM12*, were identified in C4, explaining up to 70% of the total phenotypic variance. All identified QTL exhibited dominant behavior. The confidence intervals for *qPM5*, extending to the closest flanking markers in the physical distance for the B2 and C4 populations on chromosome 5, were 25,267,104–25,725,099 bp and 25,203,821–25,678,875 bp, respectively. These overlapping intervals suggest that *qPM5* in the B2 and C4 populations can be considered the same QTL.

**Table 4:** QTL for PM resistance identified in the tree F_2_ populations

Population	QTL	Chr	Pos (cM)	a	d	LOD	*R* ^2^	Confidence interval (cM)	Flanking markers (bp)
A6	*qPM2*	2	8.9	-2.79	-2.79	94.5	93.5	8.34–10.43 [2.1]	778,333–1,142,653
B2	*qPM5*	5	92.7	-2.17	-1.28	62.8	80.1	91.63–94.67 [3.0]	25,276,104–25,725,099
C4	*qPM5*	5	89.0	-1.58	-1.10	27.7	29.3	86.45–90.58 [4.1]	25,203,821–25,678,875
	*qPM12*	12	77.4	-1.52	-1.12	28.9	31.0	75.15–78.44 [3.3]	22,493,040–22,879,440
	Total model					49.2	71.6		

Chr and Pos indicate the chromosome and peak position of QTL

Positions and length (between square brackets) of the confidence interval are indicated

Flanking markers (bp) is the confidence interval of QTL extending to the closest markers

a and d are the additive and the dominant effects of the QTL, respectively

*R*^*2*^ is the phenotypic variance explained by the QTL

### TaqMan^®^ assays for MABC

To design markers for foreground selection, the confidence interval of each QTL was extended to the closest flanking markers (Table 5 and Supplementary Table 1). The *qPM2* region, located at 8.34–10.43 cM on chromosome 2 in the A6 population, had a corresponding physical distance from 778,333 to 1,142,653 bp. The *qPM5* region, located at 91.63–94.67 cM on chromosome 5 in the B2 population and at 86.45–90.58 cM on the same chromosome in the C4 population, had overlapping physical distances of 25,267,104–25,725,099 bp for B2 and 25,203,821–25,678,875 bp for C4, with a common fragment at 25,203,821–25,725,099 bp. The *qPM12* region, identified at 75.15–78.44 cM on chromosome 12 in the C4 population, had a corresponding physical distance from 22,493,040 to 22,879,440 bp.

**Table 5:** The designed TaqMan® assays used in this study

Target	Flesh color	Code	Assay Name	Pd.cM	BC_*n*_	Context Sequence
*qPM2*	G	R1	2_382892_C-A	7.47	1	TAATAAATGTTTTAACTTTTCTAGT[C/A]TTTTTGGAAGAGGAATTGAAAGAAA
	G	F1	2_621190_A-G	9.73	1 + 2	CGTCCCTAGTTACTTCTGTTTGTTT[A/G]CCTCATTATACCACTGAAGCAACAA
	G	F2	2_1603309_A-T	19.32	1 + 2	TCGGTTCTGATAAGCAGGATGCTTC[A/T]GGAAATAAGCTTCTTCAAGATATTG
	G	R2	2_2351587_A-C	28.67	1	GGCTTGTGGAGAAGATCAACAATTC[A/C]ATTGGGCAAGACCCAAATTCTAAAT
	O	R1	2_103153_C-A	1.40	1	TCTTCTCACCCTCCATCTCATTAAC[C/A]AACTGTCAGCACCCTAAGATTCCCT
	O	F1.2	2_627533_C-T	9.78	2	GAATANTATCGTTGCATTTGATGAA[C/T]TTAAGGCTCTCCTAAGTTTAGAGCT
	O	F1.1	2_778333_T-A	10.72	1	ACGAAAATGTCACATAGCCTACAAC[T/A]CCTCCATTGACGGCATCATTCTCCC
	O	F2	2_816421_C-G	11.06	1 + 2	CCATTCTCAAATCCATCCAAAACAC[C/G]AATCCAACAAAGAACAAATAAAACC
	O	R2	2_1410652_T-C	17.03	1	CTGGTATGCTCAGTTATGCAAAGAG[T/C]GTTGCAGCTGATAACTGGTTGGCAT
*qPM5*	G/O	R1	5_24520366_C-T	86.51	1	CAACTTTCCATTTTAACTATTGGAA[C/T]GTGAGTTACTATTATTCCTTTCTTT
	G/O	F1.2	5_24941008_G-C	89.02	2	GCTTGTTCAATCTTGTGAGTTGGAT[G/C]AGATGATTTAGTTGAAGCGTGANTG
	G/O	F1.1	5_25264851_C-T	89.31	1	TTCTCCTAGTCACTTGTTTCTTACA[C/T]GAGATCTTTTGTTTCACTAGGAGGA
	G/O	F2	5_25862218_T-C	96.52	1 + 2	AACATGAAACACATCTCAATGATGA[T/C]GACGACGATGAATACCAGGCTGATT
	G/O	R2	5_26105586_T-A	99.27	1	AGGGACATCTTCATGGTTTGGTGGC[T/A]ACTTTAGCAAACTAAATTTTAGACA
*qPM12*	G	R1	12_21800139_A-G	71.69	1 + 2	CAACAAAAAATTTGTTTGATGGCCG[A/G]TAGGATTGTAACCTAATAAAAAAAC
	O	R1	12_21116734_T-G	63.61	1 + 2	GGACTATTATTCTGAACCTACCCAG[T/G]TCACAAGAGGCCCTTGTTCTTCTGA
	G/O	F1	12_22418198_A-G	78.87	1 + 2	TGAAGTAAAGTGAAAAGAAATAATC[A/G]TGTATGGCTCGCTGTTGACGTTTGT
	G/O	F2	12_23222171_G-A	84.38	1 + 2	TGATCGCCAAAGACGAGAAACTTGC[G/A]ACTTCATACCGTGATGAGAAATGGA
	G/O	R2	12_23491653_A-G	87.74	1	CCGTGGTTTGTCTATCAAGTTTTTA[A/G]AATCAACCTATGTGACAATCGCATT

The flesh colors G and O indicate the assays used for the green- or orange-fleshed recurrent parents, and G/O indicates the assay used for both recurrent parents in MABC

Codes R and F indicate the assays used for recombinant or foreground selection. The R1, R2, F1, and F2 indicate the physical position order of the TaqMan assays. The F1.1 and F1.2 indicate the F1 TaqMan assays but the different versions for their slight adjustment of the physical positions

Pd.cM indicates the predicted genetic position (cM) of the TaqMan assays through the LOESS regression models within each chromosome

BC_n_ filled with 1, 2, and 1 + 2 values indicate that the TaqMan assays were used during BC_1_, BC_2,_ or both BC_1_ and BC_2_ generations

**Fig. 7:**
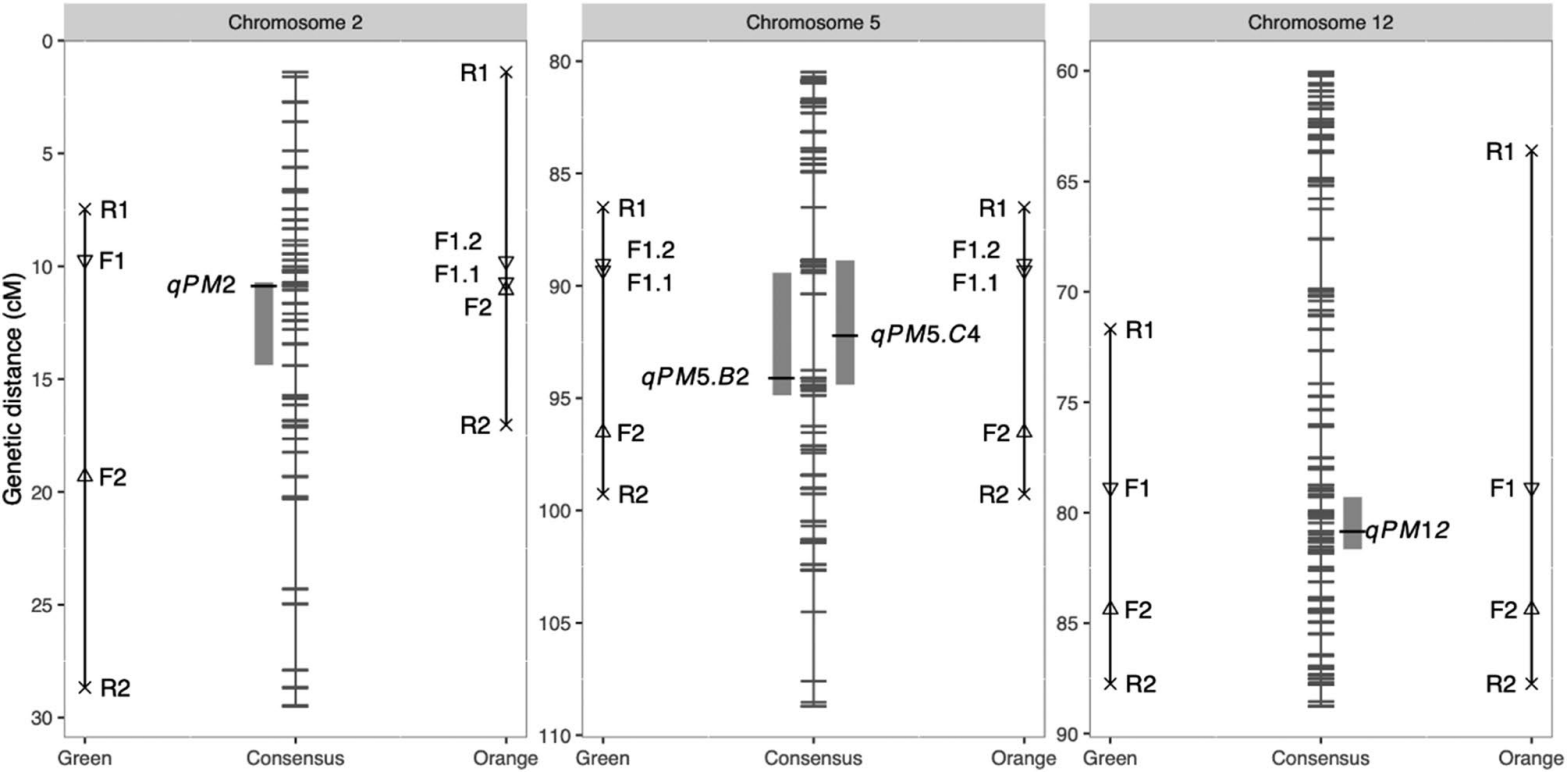
Position of the TaqMan® assays used for foreground and recombinant selection. The consensus linkage groups built from A6, B2, and C4 are shown in the middle. *qPM5.B2* and *qPM5.C4* represent the *qPM5* detected in the B2 and C4 populations, respectively. Marker positions for green- or orange-fleshed parents are indicated at the left and right sides, respectively. F1, F1.1, F1.2, F2, R1, R2 are marker names. Please refer to Table 5 and Supplementary Table 1 for detailed marker information

Accounting for polymorphic SNPs between donor and recurrent parents, and the requirements for the design tool, the designed and validated TaqMan assays are shown in Table 5. The genetic distances between bracketed foreground selection markers ranged from 0.3 to 9.6 cM for *qPM2* in the orange-fleshed parent and green-fleshed parent, respectively. The genetic distances between markers for recombinant selection and foreground selection ranged from 2.3 to 15.3 cM. The positions of markers for foreground and recombinant selection are shown in Fig. 7. Between BC_1_F_1_ and BC_2_F_1_, markers for foreground selection for *qPM2* in the orange-fleshed background and those for *qPM5* in orange- and green-fleshed backgrounds were changed from F1.1 to F1.2 (Table 5; Fig. 7).

### Generate PM resistance near-isogenic lines through MABC

#### MABC for *qPM2*

For the recombinant selection at the BC_1_F_1_ generation, 41 out of 166 (24.7%) green-fleshed individuals and 28 out of 171 (16.4%) orange-fleshed individuals showed one marker as homozygous for the recurrent parent allele and the other as heterozygous (Table 5; Figs. 1 and 7). Among the selected individuals, 9 green-fleshed and 15 orange-fleshed ones carried heterozygous genotypes for foreground markers F1, F1.1, and F2. The average RPG recovery for green- and orange-fleshed individuals was 68.0% and 66.1%, respectively. Individuals with the highest RPG recovery for green-fleshed background (#001) and orange-fleshed background (#093) were selected and crossed with the respective recurrent parent to form the BC_2_F_1_ generation. At the BC_2_F_1_ generation, a two-step selection was applied because the QTL was close to one end of chromosome 2 (Fig. 6). Among the 174 individuals in the green-fleshed BC_2_F_1_ family, 64 carried the resistance allele at the heterozygous state, while 84 out of 182 individuals in the orange-fleshed family carried heterozygous genotypes. For background selection via ddRAD sequencing, the average RPG recovery reached 86.4% and 83.0% for green- and orange-fleshed backgrounds, respectively. Individuals showing the highest RPG recovery rates, i.e., 93.6% for green-fleshed background and 89.1% for orange-fleshed background, were self-pollinated to produce the BC_2_F_2_ generation. From this step, two families were maintained: #020 and #143 for green-fleshed background and #088 and #132 for orange-fleshed background. At the BC_2_F_2_ generation, only foreground markers were used to screen 20 individuals in each family. Individuals carrying homozygous resistance genotypes were selected and self-pollinated to generate BC_2_F_3_ individuals. For each near-isogenic line, 20 BC_2_F_3_ individuals were used to evaluate the PM reaction. All the near-isogenic lines showed a DI of zero, indicating the successful introgression of PM resistance to the elite backgrounds.

#### MABC for *qPM5*

For the recombinant selection of *qPM5* at the BC_1_F_1_ generation, 20 out of 180 (11.1%) green-fleshed individuals and 12 out of 138 (8.7%) orange-fleshed individuals showed one marker as homozygous for the recurrent parents allele and the other as heterozygous (Table 5; Figs. 2 and 7). Among the selected individuals, nine green-fleshed and five orange-fleshed ones carried heterozygous genotypes for foreground markers F1.1, and F2. The average RPG recovery for green- and orange-fleshed individuals was 67.7% and 67.4%, respectively. Individuals with the highest RPG recovery for green-fleshed background (#126) and orange-fleshed background (#093) were selected and crossed with the respective recurrent parent to form the BC_2_F_1_ generation. At the second recombinant selection performed at the BC_2_F_1_ generation, 88 out of 181 individuals from the green-fleshed family and 113 out of 184 individuals from the orange-fleshed family were homozygous as the recurrent parent for the marker showing heterozygous genotype at the first recombinant selection. Among these individuals, one green-fleshed and two orange-fleshed ones were heterozygous genotypes at the foreground markers F1.2 and F2. The average RPG recovery rate was 88.3% in green-fleshed individuals and 79.6% in orange-fleshed individuals. Individuals with the highest RPG recovery, i.e., 88.3% for the green-fleshed background (#102) and 84.8% for the orange-fleshed background (#087) were selected and crossed with the respective recurrent parent to form the BC_3_F_1_ generation. At the BC_3_F_1_ generation, a two-step selection was applied since the recombinant selection was done at BC_2_F_1_. Among the 89 green-fleshed BC_3_F_1_ progeny, 37 carried the resistance allele at the heterozygous state, while 50 out of 91 orange-fleshed individuals carried heterozygous genotypes. The background selection revealed an average RPG recovery of 94.0% for the green-fleshed family and 91.1% for the orange-fleshed family. The individuals with the highest RPG recovery, i.e., 95.8% and 94.8% for green- and orange-fleshed backgrounds, respectively, were self-pollinated to generate the BC_3_F_2_ generation. Two BC_3_F_2_ families were maintained for both green- and orange-fleshed backgrounds: #051 and #080 for green-fleshed families, and #016 and #088 for orange-fleshed families. At the BC_3_F_2_ generation, only foreground markers were used to select the individuals carrying homozygous resistance alleles from 60 individuals in each family. For the green- and orange-fleshed background, selected individuals were evaluated for the DI of PM. The average DI for green- and orange-fleshed near-isogenic lines were 1.0 and 0.0, indicating that the foreground marker regions effectively improved PM resistance to the elite backgrounds.

#### MABC for *qPM12*

For the recombinant selection of *qPM12* at the BC_1_F_1_ generation, 29 out of 181 (16.0%) green-fleshed individuals and 29 out of 127 (22.8%) orange-fleshed individuals showed one marker as homozygous for the recurrent parents allele and the other as heterozygous (Table 5; Figs. 3 and 7). Among these individuals, five green-fleshed and seven orange-fleshed ones carried heterozygous genotypes for foreground markers F1 and F2. The average RPG recovery for green- and orange-fleshed individuals was 67.5% and 72.6%, respectively. Individuals with the highest RPG recovery for green-fleshed background (#159) and orange-fleshed background (#078) were selected and crossed with the respective recurrent parent to form the BC_2_F_1_ generation. At the second recombinant selection performed at the BC_2_F_1_ generation, 92 out of 182 green-fleshed individuals from the green-fleshed family and 75 out of 176 individuals from the orange-fleshed family were homozygous for the recurrent parent allele. Among these individuals, five green-fleshed and 15 orange-fleshed ones carried heterozygous genotypes for the foreground markers F1 and F2. The average RPG recovery rate was 87.0% in green-fleshed individuals and 83.6% in orange-fleshed individuals. Individuals with the highest RPG recovery, 88.2% for the green-fleshed background (#065) and 90.0% for the orange-fleshed background (#064), were selected and crossed with respective parents to form the BC_3_F_1_ generation. Among the 67 green-fleshed BC_3_F_1_ progeny, 32 carried the resistance allele at the heterozygous state, while 45 out of 90 orange-fleshed individuals carried heterozygous genotypes. The background selection revealed an average RPG recovery of 92.6% for the green-fleshed family and 93.5% for the orange-fleshed family. The individuals with the highest RPG recovery, nearing 97.1% and 96.1% for green- and orange-fleshed backgrounds, respectively, were self-pollinated to generate the BC_3_F_2_ families. Two BC_3_F_2_ families were maintained for both green- and orange-fleshed backgrounds: #022 and #044 for green-fleshed families, and #002 and #090 for orange-fleshed families. At the BC_3_F_2_ generation, only foreground markers were used to select the individuals carrying homozygous resistance alleles from 60 individuals in each family. For the green- and orange-fleshed background, selected individuals were evaluated for the DI of PM. The average DI for green-fleshed near-isogenic lines were 0.3 and 0.1, and each line showed a DI of zero in orange-fleshed backgrounds indicating that the resistance alleles of *qPM12* was effectively introgressed into the elite backgrounds.

## Discussion

In this study, we have identified three major melon PM resistance QTL located on chromosomes 2, 5, and 12 in three F_2_ populations and introduced these resistant QTL to the PM-susceptible elite lines. According to the results of PM resistance QTL in Table 4, both the A6 and B2 populations carried single PM resistance QTL, *qPM2* and *qPM5*, respectively, and the C4 population carried two, *qPM5* and *qPM12*. The phenotypic variance explained (*R*^2^) and the additive effect (a) of *qPM2* were larger than *qPM5*. While *qPM5* was accompanied by *qPM12* in the C4 population, the *R*^2^ and the LOD scores of *qPM12* were larger than *qPM5*. Although the additive effect of *qPM12* was slightly lower than *qPM5*, the average DIs of PM in the near-isogenic lines carried *qPM5* were less than those carried *qPM12* under the green-fleshed genetic background, suggesting *qPM2* and *qPM12* contribute more PM resistance than *qPM5*.

To compare the resistance QTL across different studies, we blasted the reference genome DHL92 v4 (Castanera et al. 2020) using QTL flanking marker sequences and identified candidate genes within the interval between foreground markers using the DHL92 v4 annotation (Ruggieri et al. 2018). Under the interval between foreground markers, *qPM2*, *qPM5*, and *qPM12* covered 128, 98, and 116 genes, respectively. Among this large number of genes, we will focus our discussion on candidate genes showing transcriptional or functional evidence from previous studies.

Several studies have identified PM resistance QTL overlapping *qPM2* identified in the present study, including *Pm-pxA.II* and *Pm-pxB.II* (Fukino et al. 2008), *Pm-Edisto47-1* (Ning et al. 2014), *qPM2* (Wang et al. 2016) *Pm-2F* (Zhang et al. 2013), *Pm2.1* (Haonan et al. 2020), and *Pm-II* (Kim et al. 2016). Among these QTL, *Pm-2F* and *Pm2.1* were identified through PM race 2F and *Pm-II* through race N5, while the other PM QTL on chromosome 2 were identified through the inoculation of race 1. Therefore, there may be three different QTL co-located at this region. *MELO3C015353* and *MELO3C015354* may be the candidate genes because they were annotated as disease-resistance proteins with leucine-rich repeat (LRR) domains (Haonan et al. 2020). *qPM5* identified in this study was co-localized with several PM QTL identified previously, including *Pm-AN* (Wang et al. 2011), *qPx1-5* (Branham et al. 2021), *Pm-R1-2*, and *PM-R5* (Yuste-Lisbona et al. 2011a, b). López-Martín et al. (2022) further narrowed down the region and identified the candidate genes, *MELO3C004297* and *MELO3C004311*, which encoded a branched-chain amino-acid aminotransferase-like protein and a tomato mosaic virus resistance protein N-like gene, respectively. *qPM12* identified in this study co-localized with QTL identified using different races: *Pm-pxA.XII* and *Pm-pxB.XII* (Fukino et al. 2008), *BPm12.1* (Li et al. 2017), *CmPMRl* (Cui et al. 2022), and *qPx1-12* (Branham et al. 2021), conferring resistance to race 1; *Pm-R1-2-*5 (Beraldo-Hoischen et al. 2012) to races 1, 2, and 5; *Pm-XII* to race N5 (Kim et al. 2016); *pm12.1* (Haonan et al. 2020) and *Cmpmr2F* to race 2F (Zhang et al. 2023); and *qCmPMR-12* (Cao et al. 2021) to non-specified races. López-Martín et al. (2022) further fine-mapped the QTL and identified one of the candidate genes, *MELO3C002504*, characterized as a cysteine-rich receptor-like protein kinase. Zhang et al. (2023) identified *MELO3C002403* as a candidate gene for *Cmpmr2F*. The transcription level of *MELO3C002403* in the resistant parent PI 124112 increased during PM inoculation. It encoded an allantoate amidohydrolase protein, primarily residing in the cytoplasm and cell membrane. *qPx1-12* harbored *MELO3C002392* and *MELO3C002393*, both were LRR receptor-like kinases (Branham et al. 2021). *MELO3C002434*, *MELO3C002438*, *MELO3C002439*, *MELO3C002440*, and *MELO3C002441* were Ankyrin repeat family proteins located within at least one of the following QTL: *BPm12.1*, *pm12.1*, *qCmPMR-12*, and *CmPMRl*. The parental lines for mapping *BPm12.1* and *qCmPMR-12* carried non-synonymous mutations at *MELO3C002434* and the expression level of *MELO3C002434* was significantly higher in the resistant parent after PM inoculation (Li et al. 2017; Cao et al. 2021). Therefore, it is a potential causal gene for *qPM12*. The genes *MELO3C002441* to *MELO3C002449* were located within *CmPMRl*. Among these genes, six carried non-synonymous SNPs between resistant and susceptible genotypes. In addition, *MELO3C002441*, *MELO3C002444*, and *MELO3C002448* were significantly upregulated after PM inoculation of resistant melon genotypes. Conversely, *MELO3C002446*, *MELO3C002447*, and *MELO3C002449* were downregulated during infection. *MELO3C002449* was associated with glycolytic enzyme activity but the functions of *MELO3C002446* and *MELO3C002437* remain unknown. Integrating results from different PM studies, it seems that the PM resistance QTL are predominantly clustered on chromosomes 2, 5, and 12. QTL detected through the use of different races co-localized at these three regions. Therefore, these regions may harbor multiple resistance loci and are promising targets for melon PM resistance MABC.

In this MABC study, we first focused on recombinant selection, followed by foreground and background selection. Performing recombinant selection before foreground selection at the BC_1_F_1_ generation optimized the use of low-throughput genotyping platforms. While this strategy was efficient, it could not entirely avoid the loss of target foreground genotypes due to potential double crossovers. Therefore, foreground selection was performed at each generation. The ddRAD sequencing system facilitated high-throughput genotyping for background selection in later generations, significantly increasing the intensity of selection with enhanced recovery of the RPG. Our MABC scheme at the BC_1_F_1_ generation reduces the genotyping effort by 17–28% due to the selection intensity for recombinant selection ranging from 8.7 to 24.7% in the *qPM5* orange-fleshed and *qPM2* green-fleshed MABC processes. The whole strategy was also time-effective: creating the three F_2_ populations, including the PM resistance QTL mapping, took one and a half years, followed by two years of MABC. This led to the production of near-isogenic PM resistance lines from creating the population for QTL mapping in 3.5 years.

The near-isogenic lines developed in this study are valuable resources for melon resistance breeding and genetic studies. On one hand, they can be used as parental lines to confer resistance in other elite backgrounds or allow the pyramiding of major QTL. On the other hand, they could be used for fine mapping of the three PM resistance QTL. Future research could explore the durability of resistance related to multiple minor QTL and identify additional genetic resources for resistance breeding in melon.

## Electronic supplementary material

Below is the link to the electronic supplementary material.

Supplementary Material 1

## Data Availability

The data used in this study are available from the corresponding author on reasonable request.
