# Supplementary material for: Identification of powdery mildew resistance quantitative trait loci in melon and development of resistant near-isogenic lines through marker-assisted backcrossing
Source: Bot Stud. 2024 Nov 4;65:31. doi: 10.1186/s40529-024-00435-x (PMC11534953; doi:10.1186/s40529-024-00435-x)
Supplement: Supplementary file 1 — Supplementary Material 1 [file 40529_2024_435_MOESM1_ESM.docx]

Supplementary Table 1. The sequence information of the TaqMan assays in this study.

| Assay Name | Primer Sequence (5' − 3') | Probe Sequence (5' − 3') |
| --- | --- | --- |
| 2_382892_C-A | F: GCTCAACAGTCAACAACACTACT | VIC: TTTTAACTTTTCTAGTCTTTTTG |
|  | R: GTTTCTCTTATGTGTCTTTGCTTGTAGATTATAAAT | FAM: AACTTTTCTAGTATTTTTG |
| 2_621190_A-G | F: GGTTTACATGGAGAGTTCATTAATAGCTCTTT | VIC: ACTTCTGTTTGTTTACCTCATT |
|  | R: GCTGCTATCACCTGTTTACAAGAAGTA | FAM: CTGTTTGTTTGCCTCATT |
| 2_1603309_A-T | F: TTCTGGAAGCTTCGGTTCTGATAAG | VIC: AAGCTTATTTCCTGAAGCAT |
|  | R: TCTCTGAGATAGCCATAGCCCAAT | FAM: AGCTTATTTCCAGAAGCAT |
| 2_2351587_A-C | F: CTGTAGGCTTGTGGAGAAGATCA | VIC: TGCCCAATTGAATTGT |
|  | R: TCAAATCCATAAATATCCAAAACTCCAATCAATG | FAM: CCCAATGGAATTGT |
| 2_103153_C-A | F: CCCTTCTTCTCACCCTCCATCT | VIC: CTGACAGTTGGTTAATG |
|  | R: TCGTGGAAATATATGGTAGGAGTAGTAAGG | FAM: CTGACAGTTTGTTAATG |
| 2_627533_C-T | F: ATTTATCGTACTCTTTCCACTTCTTGTGAAT | VIC: AGAGCCTTAAGTTCATC |
|  | R: CTAATTGAGAATGGATTCTAGCTCTAAACTTAGG | FAM: AGAGCCTTAAATTCATC |
| 2_778333_T-A | F: CCCCAAGCTCTTACGAAAATGTCA | VIC: CATAGCCTACAACTCCTCCA |
|  | R: GGGAGAATGATGCCGTCAA | FAM: TAGCCTACAACACCTCCA |
| 2_816421_C-G | F: CCCACTTCCCATTCTCAAATCCAT | VIC: TTTGTTGGATTGGTGTTTT |
|  | R: GCTGTTGGGTTTGTTGGGTTTTATT | FAM: TTGTTGGATTCGTGTTTT |
| 2_1410652_T-C | F: TCCAATAATTTTTCTCTGGTATGCTCAGTT | VIC: CTGCAACACTCTTTG |
|  | R: ATCCATCAATGCCAACCAGTTATCA | FAM: TGCAACGCTCTTTG |
| 5_24520366_C-T | F: ATAACAAATATTCTTGAACTTTTCAACTTTCCA | VIC: AATAGTAACTCACGTTCCAAT |
|  | R: TTTTTATAAAATTAAGTGTTTGATCAGGAAAGAAAGGAA | FAM: AATAGTAACTCACATTCCAAT |
| 5_24941008_G-C | F: CCTGTTGTAGGCTTGTTCAATCTTG | VIC: TGAGTTGGATGAGATGAT |
|  | R: CCAAACAAGCCCATTTTGCA | FAM: TGAGTTGGATCAGATGAT |
| 5_25264851_C-T | F: AAAATCTTGGTTTTCTCCTAGTCACTTGT | VIC: CAAAAGATCTCGTGTAAGAA |
|  | R: CTCTTTTCTTCTTTCATCCTCCTAGTGA | FAM: ACAAAAGATCTCATGTAAGAA |
| 5_25862218_T-C | F: CTCAACCCTCTGATTCTGATGAACA | VIC: ATCGTCGTCATCATCA |
|  | R: TTCATTGTTTTGTGCATCTTCTTGGT | FAM: ATCGTCGTCGTCATCA |
| 5_26105586_T-A | F: GGTAGTGTGTAATAAACCTAGGGACATC | VIC: TGGTTTGGTGGCTACTTT |
|  | R: CCTGGGATCTTCCAATCAAAGTTGT | FAM: TGGTGGCAACTTT |
| 12_21800139_A-G | F: TGATTCTTGGAGTTGTGGTAAAGCA | VIC: TGATGGCCGATAGGATT |
|  | R: GTAATCTATATTACAAATCGGATACAACTTATTGAAATTCAAAT | FAM: ATGGCCGGTAGGATT |
| 12_21116734_T-G | F: TGTTTGTTTTAGTTCTTGAGGTTTGGACTA | VIC: AACCTACCCAGTTCACAAG |
|  | R: GAAAGCTCCCATACTCAGAAGAACA | FAM: CCTACCCAGGTCACAAG |
| 12_22418198_A-G | F: GCTCAGAATCGGGTATTGTTGTTGA | VIC: AGCCATACATGATTATTT |
|  | R: CGTGGAACAGTTAACATGCATATTTTGA | FAM: CCATACACGATTATTT |
| 12_23222171_G-A | F: TGACAACATTTTGATCGCCAAAGAC | VIC: AGAAACTTGCGACTTCA |
|  | R: CTCAACTTCAAACTTTGGCTTCCAT | FAM: AAACTTGCAACTTCA |
| 12_23491653_A-G | F: ACCTTTCATAAATGATAGTCCGTGGTT | VIC: CATAGGTTGATTTTAAAAAC |
|  | R: GCTACATGCTAGAATGCGATTGTCA | FAM: TAGGTTGATTCTAAAAAC |

F and R indicate the forward and reverse primer; VIC and FAM indicate the two fluorescent dyes labeled on the minor groove-binding probes.
